# Modeling and analysis of high aspect ratio wing considering random structural parameters

**DOI:** 10.1038/s41598-021-95187-0

**Published:** 2021-08-02

**Authors:** Bangsheng Fu, Ya Yang, Hui Qi, Jiangtao Xu, Shaobo Wang

**Affiliations:** 1 Department of Aerospace Engineering, Harbin Engineering University, Harbin, 150001 China; 2 School of Electronics and Information Engineering, Zhongyuan University of Technology, Zhengzhou, 450001 China; 3 System Design Institute of Hubei Aerospace Technology Academy, Wuhan, 430040 China

**Keywords:** Engineering, Aerospace engineering

## Abstract

With the application of advanced composite materials in High-Aspect-Ratio wings (HARW), the randomness of structural parameters, such as elastic modulus and Poisson's ratio, is enhanced. Hence, in order to explore the whole picture of aeroelastic problems, it is of great significance to study the role of random structural parameters in aeroelastic problems. In this paper, the dynamic response of flexible HARW considering random structural parameters is analyzed. An aeroelastic model of a one-dimensional cantilevered Euler–Bernoulli beam considering aerodynamic forces acting on the wing is established based on Hamilton's principle. Adopted the idea of simplifying calculation, the effect of random structural parameters is analyzed. Then, considering the elastic modulus and torsional stiffness as continuously one-dimensional random field functions, and discretized by local method. The first and second order recursive stochastic nonlinear finite element equations of wing are derived by using perturbation method. Based on it, statistical expression of aeroelastic effects of the wing is derived. Monte Carlo method is adopted to verify the effectiveness of the method. Numerical simulations indicate that the method proposed can well mirror the statistical characteristics of aeroelastic response.

## Introduction

Aeroelastic problems of flexible HARW are of great concern in developing High-Altitude Long-Endurance aircraft, solar-powered aircraft and heavy rotorcraft^1^. These wings have the characteristics of lightweight and great flexibility. The flexibility of long-span wings makes it possible to generate large deflections during in flight. It is unlikely to get the accurate aeroelastic results by the conventional linear aeroelastic analysis method. Studies has been shown that the aeroelastic results of the nonlinear analysis are different from those of linear analysis^2–4^. Due to the development of modeling technology of complex nonlinear systems and the development of mathematical calculation tools, nonlinear aeroelastic problems have been greatly developed in the past two decades^5^.

Patil^5, 6^ established a complete aircraft model of high altitude long endurance aircraft and studied its nonlinear aeroelasticity. Significant changes can occur in the wing’s natural frequencies because the tip displacement function is very closely related to the flutter speed. Tang^7^ carried out theoretical analysis and wind tunnel experimental study on geometric nonlinearity’s influence on aeroelastic characteristics of flexible HARW. The effects of the geometric structural nonlinearity are determined by the ratio of the flap and chord wise bending stiffness. When the ratio is relatively small, the boundary of flutter instability has a little change due to the structural nonlinearity and preflutter static deformation. Garcia^8^ analyzed the flexible HARW’s aeroelastic characteristics at transonic speeds. The results show that the nonlinear static aeroelastic characteristics of flexible wing with high aspect ratio are different from those of linear aeroelastic response in transonic flow. These differences are owing to the coupling of large transverse bending deflections with drag force, and the kinematic effects of bent wing structures at transonic speeds. Tang^9^ studied the effect of geometric nonlinearity on the limit cycle oscillations (LCOS) of flexible HARW by both experiment and theoretical calculation. The research shows that the LCO hysteretic response is usually dependent on a delicate balance between stall aerodynamics and the structural nonlinear forces. Shearer^10^ presented a six-degree-of-freedom coupled vehicle dynamics model, which is an upgraded version of the high-aspect ratio lifting surface structure model based on low-order nonlinear strain. The dynamic responses of nonlinear rigid body coupled nonlinear rigid body and fully nonlinear aeroelastic aircraft in pitch, roll and yaw flight are analyzed by using this model. The simulation results show that the modeling of nonlinear structure is of great significance for high altitude long-endurance flexible aircraft. Su^11, 12^ used the finite element framework based on low-order nonlinear strain to model the high-flexible wings and analyzed the nonlinear aeroelastic coupling model.

With the development of aerospace technology, many advanced concepts and new materials and structures have been applied to the next generation of aircraft’s design and manufacture. But at the same time, many new problems have arisen. One of them is the use of composite materials, which leads to many random structural parameters in the aircraft manufacturing process^13^. Physical properties of structure materials, such as Young's modulus, Poisson's ratio, etc., structural properties or even nonlinearities^14, 15^ can be considered as random factors, which can be represented as random field distribution functions. Due to these factors, the response of structure under excitation will not be linear, but appears as time and field functions of random variables.

In traditional way, aeroelastic analysis is carried out under the condition that the random structural parameters are regarded as determining values^2–12, 16–18^. However, in reality, the aeroelastic response of wing will be affected by random structural parameters. It becomes even more problematic when nonlinearities are involved because some non-negligible nonlinear factors will cause serious instability. Based on classical finite element method, stochastic finite element method (SFEM) is an extension based on the stochastic framework, which is used to solve stochastic problems with finite elements with random properties^19^. It can deal with both the structural parameters stochastic problems, and the input stochastic problems^20, 21^. By analyzing the microstructure model of composites, Stefanou^22^ obtained the statistical information of the probability distribution and correlation of the composite properties at the micro scale. Based on SFEM, the macro and micro mechanical responses of the composite structure were calculated, and the influence of the micro properties on the response was studied. Sepahvand^23^ regards the damping coefficient of fiber reinforced composites as a random variable, describes the random characteristics of structural damping by generalized polynomial chaotic expansion method, and analyzes the damping vibration by spectral stochastic finite element method. Zhou^24^ combined the homogenization method and stochastic finite element method, considered the micro and macro uncertainties of material properties and ply angle, proposed a multi-scale method to analyze the response and reliability of composite structures, and used Monte Carlo Simulation (MCS) simulation to verify the method. Chang^25^ established a bridge-vehicle model based on SFEM, considering the random material properties and moving loads, analyzed the statistical dynamics. Based on SFEM concept Huh^26^ proposed an efficient algorithm to evaluate the seismic risk of nonlinear structures, seismic excitation and other uncertainty parameters are incorporated for modeling. Examples showed the validity of the method for real nonlinear structures evaluation subjected to seismic loadings. Stochastic seismic analysis of long-span bridges with Carbon fibre reinforced polymer cables (CFRP) are presented based on SFEM and MCS by vdar^27^, geometric nonlinear effects and material uncertainties are considered, the feasibility of using CFRP cables in long-span bridge under seismic excitation is discussed. Niranjan^28^ modeled diverse material properties of functionally graded materials and piezoelectric materials with volume fraction exponent as independent random variables. SFEM was used to obtain the statistics of nonlinear natural frequency response of second-order. Xu^29^ uses SFEM to model and verify the aeroelasticity of slender aircraft with stochastic factors. The results show that the established model has good statistical characteristics of aeroelastic response.

SFEM is adopted to study the structural reliability, disaster evaluation and composite properties. And the aeroelastic response of HARW with geometric nonlinearity or SFEM are studied by some existing literatures, but most of these articles consider the random parameters as determining value. The aeroelasticity considering random parameters of HARW has not been reported.

On the basis of the previous study, SFEM is used to study the effect of the random structural parameters on the aeroelastic response of HARW considering geometric nonlinearity in this paper. Firstly, the dynamics model of the cantilevered wing is established under the action of the unsteady aerodynamic force. For the purpose of simplifying the calculation, the elastic modulus as well as torsional stiffness are selected as random structural parameters. Then, these two parameters are considered as random field functions, using local average method to discretize these functions in order to obtain the results of aeroelastic response, the SFEM is adopted as the solver for the model, and the comparisons between the SFEM and Monte Carlo method are given. Finally, under the random factors, the aeroelastic response of the wings is studied.

## Flexible body dynamics model

**Figure 1:**
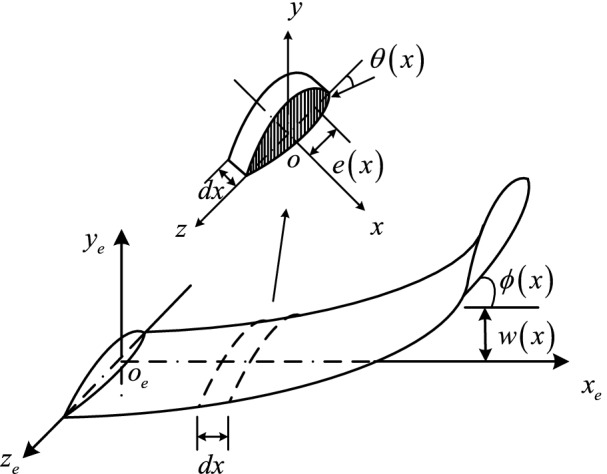
Definition of coordinate system.

HARW in air vehicle are subject to gravity, aerodynamic force, and these forces will result in multiple elastic deformation forms of the structure. Among them, bending and twisting are the most basic forms. In order to obtain a simplified model, we regard the flexible HARW as a cantilevered Euler–Bernoulli beam^4^. The associated coordinate system is defined as shown in Fig. 1. This paper assumes that the torsion centers of each cross-section of the wing are in the same straight line, called elastic axis. Each cross-section of the wing is twisted around the elastic axis, which is perpendicular to the wing root section. The distance between the aerodynamic center and the elastic axis is $e\left( x \right)$, $l$ is half span.

The wing’s elastic deformation is defined in the coordinate system $o_{e} x_{e} y_{e} z_{e}$, whose origin at the torsion center of the wing root cross-section. The $o_{e} x_{e}$-axis is pointing in the elastic axis direction. The $o_{e} y_{e}$-axis is located at the wing root cross-section and is perpendicular to the chord. $w(x)$ and $\phi (x)$ are the displacement and rotation angle of the elastic deformation of the wing in the $o_{e} x_{e} y_{e}$ plane. $\theta (x)$ represents the torsional deformation of the cross-section. $oxyz$ is a moving coordinate system with the origin fixed at the torsion center of the wing’s cross section. The coordinate system is defined analogous to the $o_{e} x_{e} y_{e} z_{e}$.

The elastic potential energy can be expressed as

1 $$U = \frac{1}{2}\int_{V} {\user2{\sigma \varepsilon }dV} = \frac{1}{2}\int_{V} {{\varvec{\varepsilon}}^{T} \user2{D\varepsilon }dV}$$

Here, $${\varvec{\sigma}} = \left[ {\begin{array}{*{20}c} {\sigma_{x} } & {\tau_{yz} } \\ \end{array} } \right]^{T} ,{\varvec{\varepsilon}} = \left[ {\begin{array}{*{20}c} {\varepsilon_{x} } & {\gamma_{yz} } \\ \end{array} } \right]^{T}$$.

$\sigma_{x}$ and $\tau_{yz}$ are normal stress and shear stress respectively;$\varepsilon_{x}$, $\gamma_{yz}$ is the corresponding strain; ${\varvec{D}}$ is an elastic matrix.

**Figure 2:**
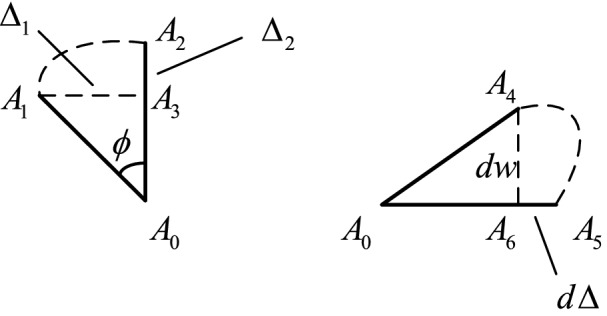
Deformation relationship.

As shown in Fig. 2, micro-segments *A*_0_*A*_2_ and *A*_0_*A*_5_ are taken in the cross-section. The length is $d\delta$.$\Delta_{1}$, $\Delta_{2}$ respectively represent the displacement in the $x$ direction and the $y$ direction due to the rotation angle of the cross-section. $d\Delta$ represents the displacement in the $x$ direction caused by the bending deformation $dw$.

It is assumed that the length of the micro-segment after deformation remains unchanged. $\Delta_{1}$, $\Delta_{2}$ can be expressed as:

2 $$\begin{array}{*{20}c} {\Delta_{1} = A_{0} A_{1} \sin \phi = d\delta \sin \phi } \\ {\Delta_{2} { = }A_{0} A_{2} - A_{0} A_{3} = d\delta \left( {1 - \cos \phi } \right)} \\ \end{array}$$

Taylor expansion of the above formula yields:

3 $$\begin{array}{*{20}c} {\Delta_{1} = - d\delta \frac{\partial w}{{\partial x}}} \\ {\Delta_{2} = - \frac{1}{2}d\delta \left( {\frac{\partial w}{{\partial x}}} \right)^{2} } \\ \end{array}$$

From Fig. 2, $d\Delta$ can be expressed as:

4 $$d\Delta = \left( {1 - \sqrt {1 - \left( {\frac{\partial w}{{\partial \delta }}} \right)^{2} } } \right)d\delta$$

After Taylor's expansion,

5 $$d\Delta = - \frac{1}{2}\left( {\frac{\partial w}{{\partial \delta }}} \right)^{2} d\delta$$

Assuming torsional deformation of the micro-segment is small, the displacement at any point $\left( {0,y_{e} ,z_{e} } \right)$ of the wing cross-section is:

6 $$\begin{array}{*{20}c} {\overline{u} = u - y_{e} \frac{\partial w}{{\partial x}} - \int_{0}^{x} {\frac{1}{2}\left( {\frac{\partial w}{{\partial x}}} \right)^{2} } d\delta } \\ {\overline{w} = w - \frac{1}{2}y_{e} \left( {\frac{\partial w}{{\partial x}}} \right)^{2} + z_{e} \theta } \\ \end{array}$$

The relationship between strain and displacement can be expressed as follows:

7 $$\begin{array}{*{20}c} {\varepsilon_{x} = \frac{{\partial \overline{u}}}{\partial x} + \frac{1}{2}\left[ {\left( {\frac{{\partial \overline{u}}}{\partial x}} \right)^{2} + \left( {\frac{{\partial \overline{w}}}{\partial x}} \right)^{2} } \right]} \\ {\gamma_{yz} = \frac{1}{2}\left( {\frac{{\partial \overline{w}}}{\partial z} + \frac{{\partial \overline{u}}}{\partial y}\frac{{\partial \overline{u}}}{\partial z} + \frac{{\partial \overline{w}}}{\partial y}\frac{{\partial \overline{w}}}{\partial z}} \right)} \\ \end{array}$$

The kinetic energy generated by the deformed beam is as follows:

8 $$T = \frac{1}{2}\int_{V} {dm\left( {\dot{\overline{u}}^{2} + \dot{\overline{w}}^{2} } \right)dV}$$

Here, $dm$ is mass density.

The work $\delta W_{nc}$ done by the external force is

9 $$\delta W_{nc} = \int_{0}^{l} {\left( {d{\varvec{L}}_{w} \delta \overline{w} + d{\varvec{M}}_{w} \delta \theta } \right)} dx$$

where ${\varvec{L}}_{w}$, ${\varvec{M}}_{w}$ is lift and moment, respectively.

Substitute Eqs. (1), (8), and (9) into Eq. (10), the flexible wing dynamical model can be obtained as

10 $$\delta \int_{{t_{1} }}^{{t_{2} }} {\left( {T - U_{c} } \right)} dt + \int_{{t_{1} }}^{{t_{2} }} {\delta W_{nc} dt} = 0$$

## Unsteady aerodynamics

Although the quasi-steady approximate method is simple, and its accuracy is poor in dynamic aeroelastic analysis of HARWs. However, the aerodynamic model with higher precision would increase the difficulty of obtaining partial derivatives of aerodynamic force to structural random paramaters. At the same time, more accuracy aerodynamic model would bring in more uncertainty and nonlinearity, which makes it more difficult to study the influence of structural random factors on the model, and further makes the study of the influence of structural random factors on the aeroelastic response more complicated. Since the research focus of this paper is mainly on the influence of structural random factors, in order to simplify the calculation, we adopt quasi-steady approximate method to carry out the research while ensuring the certain accuracy of the aerodynamic model.

${\varvec{L}}_{w}$, ${\varvec{M}}_{w}$ in Eq. (9) are distributed forces. For the unsteady aerodynamic forces and moments, Theodorsen^30^ proposed the theory of unsteady aerodynamics for a thin airfoil undergoing small oscillations in incompressible flow. This method is used to study the amplitude and phase problems of sinusoidal unsteady aerodynamic force at different reduce frequencies. Peters^31^ proposed a finite state aerodynamic theory for a two-dimensional thin airfoil operating in inviscid and incompressible flow. This theory is derived directly from potential flow theory with no assumptions on the time history of airfoil motions. The aerodynamic states are the coefficients of a set of induced-flow expansions. Based on^30^, for a particular reduced frequency, the lift and moment per unit span for a wing about its aerodynamic center are given by

11 $${\varvec{L}}_{w} = \rho v^{2} \left( {{\varvec{L}}_{y} y_{i} + {\varvec{L}}_{{\dot{y}}} \frac{{b\dot{y}_{i} }}{v} + {\varvec{L}}_{\alpha } b\alpha + {\varvec{L}}_{{\dot{\alpha }}} \frac{{b^{2} \dot{\alpha }}}{v}} \right)$$

12 $${\varvec{M}}_{w} = \rho v^{2} \left( {{\varvec{M}}_{y} by_{i} + {\varvec{M}}_{{\dot{y}}} \frac{{b^{2} \dot{y}_{i} }}{v} + {\varvec{M}}_{\alpha } b^{2} \alpha + {\varvec{M}}_{{\dot{\alpha }}} \frac{{b^{3} \dot{\alpha }}}{v}} \right)$$

where $\rho$ is air density; $y_{i}$ is the displacement of the motion in the heave;$v$ is the speed of the aircraft; $b$ is the semi-chord; $\alpha$ is the angle of attack; ${\varvec{L}}_{\alpha }$ is the lift curve slope. ${\varvec{L}}_{y}$, ${\varvec{M}}_{y}$ etc. are the aerodynamic derivatives of dimensionless. These derivatives are expressed by the normalized displacement and velocity, for example

$${\varvec{L}}_{y} = \frac{{\partial C_{L} }}{{\partial \left( {{{y_{i} } \mathord{\left/ {\vphantom {{y_{i} } b}} \right. \kern-\nulldelimiterspace} b}} \right)}}\user2{,L}_{{\dot{y}}} = \frac{{\partial C_{L} }}{{\partial \left( {{{\dot{y}_{i} } \mathord{\left/ {\vphantom {{\dot{y}_{i} } v}} \right. \kern-\nulldelimiterspace} v}} \right)}}$$

where $C_{L}$ is lift coefficient.

Taking the quasi-steady assumption, the reduced frequency $k \to 0$, the lift and pitching moment about the elastic axis become

13 $${\varvec{L}}_{w} = \frac{1}{2}\rho v^{2} c{\varvec{L}}_{\alpha } \left( {\alpha + \frac{{\dot{y}_{h} }}{v}} \right)$$

14 $${\varvec{M}}_{w} = \frac{1}{2}\rho v^{2} ec{\varvec{L}}_{\alpha } \left( {\alpha + \frac{{\dot{y}_{h} }}{v}} \right)$$

Here, $c$ is the chord.

Due to the assumption, the shortage of the quasi-steady assumption is low precision. Consequently, the unsteady aerodynamic derivative term should be retained in the flutter analysis as it has been proved that it has an important influence on the unsteady aerodynamic behavior^32^, so Eq. (14) can be rewritten as:

15 $${\varvec{M}}_{w} = \frac{1}{2}\rho v^{2} c\left[ {e{\varvec{L}}_{\alpha } \left( {\alpha + \frac{{\dot{y}_{h} }}{v}} \right) + {\varvec{M}}_{{\dot{\alpha }}} \frac{{c^{2} \dot{\alpha }}}{4v}} \right]$$

After simplifying and adding unsteady aerodynamic derivative term, Eqs. (13) and (15) are the lift and pitching moment about the elastic axis, respectively. In Eq. (15), aeroelasticity derivative ${\varvec{M}}_{{\dot{\alpha }}}$ is related to reduced frequency $k$, in this study, based on the equation of motion and the given flow speed, an iterative procedure is adopted for reduced frequency calculation^33^, the calculated converge value is 0.13 in this study.

## SFEM discretization

After establishing the model of a flexible HARW under unsteady aerodynamics, the SFEM will be used to solve the model. Firstly, the Hermite element is used to discretize the dynamic equations above. The transverse displacement within the element $${\varvec{u}}^{e} \left( {x,t} \right) = \left[ {\begin{array}{*{20}c} {w\left( {x,t} \right)} & {\theta \left( {x,t} \right)} \\ \end{array} } \right]^{T}$$ can be indicated as

16 $${\varvec{u}}^{e} \left( {x,t} \right) = \user2{N\chi }^{{\varvec{e}}}$$

where ${\varvec{\chi}}^{{\varvec{e}}}$ is the unit node’s displacement vector, and ${\varvec{N}}$ is the shape function vector. By Hamilton’s principle and FEM, the equation of motion can be expressed as:

17 $${\varvec{M}}\left\{ {\user2{\ddot{\chi }}} \right\} + {\varvec{C}}\left\{ {\dot{\user2{\chi }}} \right\} + \left( {{\varvec{K}}_{l} + {\varvec{K}}_{n} } \right)\left\{ {\varvec{\chi}} \right\} = {\varvec{Q}}\left( t \right)$$

where ${\varvec{M}}$ denotes the deduced mass matrix, ${\varvec{C}}$ represents the deduced damping matrix. ${\varvec{K}}_{l}$ and ${\varvec{K}}_{n}$ indicate the linear and the nonlinear stiffness matrix of the structure element, respectively. ${\varvec{Q}}(t)$ is the load vector.

**Figure 3:**
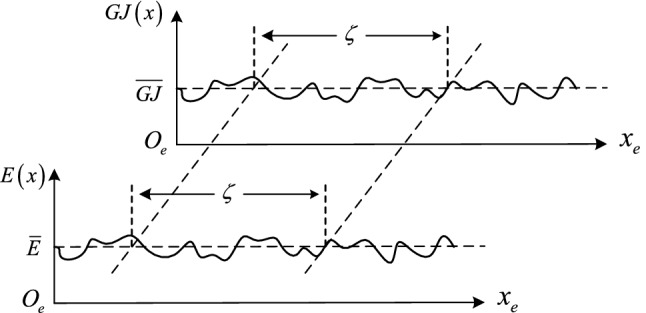
Random field.

In this study, take the elastic modulus and the torsional stiffness as random fields $\tilde{E}\left( x \right)$ and $\widetilde{GJ}\left( x \right)$, all two random fields are regard as one dimension and stationarily. $\overline{E}$ and $\overline{GJ}$ are their mean values. The variance are $\sigma_{E}^{2}$ and $\sigma_{GJ}^{2}$. By using the local average method^34^, $\tilde{E}\left( x \right)$ and $\widetilde{GJ}\left( x \right)$ can be discretized as follows:

18 $$\tilde{E}_{i} = \frac{1}{{l_{e} }}\int_{0}^{{l_{e} }} {\tilde{E}\left( x \right)} dx$$

19 $$\widetilde{GJ}_{i} \left( {l_{e} } \right) = \frac{1}{{l_{e} }}\int_{0}^{{l_{e} }} {\widetilde{GJ}\left( x \right)} dx$$

where, $l_{e}$ is the length of the unit, $\tilde{E}_{i} \left( {l_{e} } \right)$ and $\widetilde{GJ}_{i} \left( {l_{e} } \right)$ are the local average random fields. For random fields of arbitrary form, the random variables at any two positions are correlated. The strength of correlation determined by the random ield’s properties. For describing the random field’s correlation characteristics, we can adopt the correlation equation as:

20 $$\tau \left( \zeta \right) = e^{ - b\left| \zeta \right|} cos\left( {b\zeta } \right)$$

where, $\delta u$ is the correlation distance of $\tilde{E}\left( x \right)$^35^, $b = {1 \mathord{\left/ {\vphantom {1 {\delta u}}} \right. \kern-\nulldelimiterspace} {\delta u}}$. As seen in Fig. 3, $\tau \left( \zeta \right)$ is defined as the correlation at the location of $\zeta$.

The variance reduction function $\Omega \left( {l_{e} } \right)$, which describes the relationship between discrete random variables and random field variance, can be written as

21 $$\begin{aligned} \Omega \left( {l_{e} } \right) &= \frac{1}{{l_{e}^{2} }}\int_{0}^{{l_{e} }} {\int_{0}^{{l_{e} }} {\tau \left( {x_{1} - x_{2} } \right)dx_{1} dx_{2} } } \\ &= \frac{\delta u}{{l_{e} }} - \left( {\frac{\delta u}{{l_{e} }}} \right)^{2} e^{{{{ - l_{e} } \mathord{\left/ {\vphantom {{ - l_{e} } {\delta u}}} \right. \kern-\nulldelimiterspace} {\delta u}}}} sin\left( {\frac{{l_{e} }}{\delta u}} \right) \\ \end{aligned}$$

From Eqs. (18–21), The expression of the random field can then be rewritten as a vector form ${\varvec{\varTheta}}$. Using Taylor’s series expansion near the mean value of ${\varvec{\varTheta}}$ (that is $\overline{\user2{\Theta }}$ ), reserving first order term, the following equation can be obtained

22 $${\varvec{\chi}}\left( {{\varvec{\varTheta}},t} \right) = \overline{\user2{\chi }}\left( t \right) + \sum\limits_{i = 1}^{q} {\overline{\user2{\chi }}_{{\Theta_{i} }} \left( t \right)} \Delta \Theta_{i}$$

23 $$\begin{gathered} {\varvec{M}}\left( {\user2{\Theta ,}t} \right) = \overline{\user2{M}}\left( t \right) + \sum\limits_{i = 1}^{q} {\overline{\user2{M}}_{{\Theta_{i} }} \left( t \right)} \Delta \Theta_{i} \hfill \\ \begin{array}{*{20}c} {{\varvec{C}}\left( {\user2{\Theta ,}t} \right) = \overline{\user2{C}}\left( t \right) + \sum\limits_{i = 1}^{q} {\overline{\user2{C}}_{{\Theta_{i} }} \left( t \right)} \Delta \Theta_{i} } \\ {{\varvec{K}}_{l} \left( {\user2{\Theta ,}t} \right) = \overline{\user2{K}}_{l} \left( t \right) + \sum\limits_{i = 1}^{q} {\left( {\overline{\user2{K}}_{l} } \right)_{{\Theta_{i} }} \left( t \right)} \Delta \Theta_{i} } \\ {{\varvec{K}}_{n} \left( {\user2{\Theta ,}t} \right) = \overline{\user2{K}}_{n} \left( t \right) + \sum\limits_{i = 1}^{q} {\left( {\overline{\user2{K}}_{n} } \right)_{{\Theta_{i} }} \left( t \right)} \Delta \Theta_{i} } \\ {{\varvec{Q}}\left( {{\varvec{\varTheta}},t} \right) = \overline{\user2{Q}}\left( t \right) + \sum\limits_{i = 1}^{q} {\overline{\user2{Q}}_{{\Theta_{i} }} \left( t \right)} \Delta \Theta_{i} } \\ \end{array} \hfill \\ \end{gathered}$$

where, $\overline{\user2{\Theta }}$ ,$\overline{\user2{\chi }}\left( t \right)$ stand for the corresponding mean value, $\Delta \Theta$ stands for a random variable with a zero mean. and $\overline{\user2{\chi }}_{{\Theta_{i} }} \left( t \right)$ denotes the first partial derivative of $\overline{\user2{\chi }}\left( t \right)$ with respect to $\Theta_{i}$ estimated at $\overline{\user2{\Theta }}$, respectively. $q$ is the number order of random parameters. Variables in Eq. (23) are similar to the above.

The form of the first partial derivative of the stiffness matrix with respect to the random parameters is

24 $$\begin{aligned} \overline{\user2{K}}_{{\tilde{\Theta }_{Ij} }} \left( t \right) &= \frac{{\partial \overline{\user2{K}}\left( {\tilde{\user2{\Theta }}_{I} } \right)}}{{\partial \tilde{\Theta }_{Ij} }} = {\varvec{t}}^{T} \frac{{\partial \overline{\user2{K}}_{e} \left( {\tilde{\user2{\Theta }}_{I} } \right)}}{{\partial \tilde{\Theta }_{Ij} }}{\varvec{t}} \hfill \\ &= {\varvec{t}}^{T} \frac{\partial }{{\partial \tilde{\Theta }_{Ij} }}\left( \begin{gathered} \int_{{V_{0} }} {{\varvec{B}}_{{\varvec{L}}}^{T} {\varvec{DB}}_{{\varvec{L}}} } dV_{0} + \frac{1}{2}\int_{{V_{0} }} {{\varvec{B}}_{{\varvec{L}}}^{T} {\varvec{D}}\left( {{\varvec{AG}}} \right)} dV_{0} + \hfill \\ \int_{{V_{0} }} {\left( {{\varvec{AG}}} \right)^{T} {\varvec{DB}}_{{\varvec{L}}} } dV_{0} + \frac{1}{2}\int_{{V_{0} }} {\left( {{\varvec{AG}}} \right)^{T} {\varvec{DAG}}} dV_{0} \hfill \\ \end{gathered} \right){\varvec{t}} \hfill \\ &= \left( {\begin{array}{*{20}c} {{\varvec{t}}^{T} \int_{{V_{0} }} {\frac{{\partial {\varvec{B}}_{{\varvec{L}}}^{T} {\varvec{DB}}_{{\varvec{L}}} }}{{\partial \tilde{\Theta }_{Ij} }}} dV_{0} {\varvec{t}}} \\ { + \frac{1}{2}{\varvec{t}}^{T} \int_{{V_{0} }} {\frac{{\partial {\varvec{B}}_{{\varvec{L}}}^{T} {\varvec{D}}}}{{\partial \tilde{\Theta }_{Ij} }}\left( {{\varvec{AG}}} \right) + {\varvec{B}}_{{\varvec{L}}}^{T} {\varvec{D}}} \left( {\left( {\frac{{\partial {\varvec{A}}}}{{\partial {\varvec{d}}_{{\varvec{e}}} }}\frac{{\partial {\varvec{d}}_{{\varvec{e}}} }}{{\partial \tilde{\Theta }_{Ij} }}} \right){\varvec{G}} + {\varvec{A}}\frac{{\partial {\varvec{G}}}}{{\partial \tilde{\Theta }_{Ij} }}} \right)dV_{0} {\varvec{t}}} \\ { + {\varvec{t}}^{T} \int_{{V_{0} }} {\left( {\frac{{\partial {\varvec{G}}^{T} }}{{\partial \tilde{\Theta }_{Ij} }}{\varvec{A}} + {\varvec{G}}^{T} \left( {\frac{{\partial {\varvec{A}}}}{{\partial {\varvec{d}}_{{\varvec{e}}} }}\frac{{\partial {\varvec{d}}_{{\varvec{e}}} }}{{\partial \tilde{\Theta }_{Ij} }}} \right)} \right){\varvec{DB}}_{{\varvec{L}}} } + \left( {{\varvec{AG}}} \right)^{T} \frac{{\partial {\varvec{DB}}_{{\varvec{L}}} }}{{\partial \tilde{\Theta }_{Ij} }}dV_{0} {\varvec{t}}} \\ {\begin{array}{*{20}c} { + \frac{1}{2}{\varvec{t}}^{T} \int_{{V_{0} }} {\left( {\frac{{\partial {\varvec{G}}^{T} }}{{\partial \tilde{\Theta }_{Ij} }}{\varvec{A}} + {\varvec{G}}^{T} \left( {\frac{{\partial {\varvec{A}}}}{{\partial {\varvec{d}}_{{\varvec{e}}} }}\frac{{\partial {\varvec{d}}_{{\varvec{e}}} }}{{\partial \tilde{\Theta }_{Ij} }}} \right)} \right){\varvec{DAG}}} dV_{0} {\varvec{t}}} \\ { + \frac{1}{2}{\varvec{t}}^{T} \int_{{V_{0} }} {\left( {{\varvec{AG}}} \right)^{T} \left( {\frac{{\partial {\varvec{D}}}}{{\partial \tilde{\Theta }_{Ij} }}{\varvec{AG}} + {\varvec{D}}\left( {\left( {\frac{{\partial {\varvec{A}}}}{{\partial {\varvec{d}}_{{\varvec{e}}} }}\frac{{\partial {\varvec{d}}_{{\varvec{e}}} }}{{\partial \tilde{\Theta }_{Ij} }}} \right){\varvec{G}} + {\varvec{A}}\frac{{\partial {\varvec{G}}}}{{\partial \tilde{\Theta }_{Ij} }}} \right)} \right)} dV_{0} {\varvec{t}}} \\ \end{array} } \\ \end{array} } \right) \hfill \\ \end{aligned}$$

Substitute Eqs. (22, 23) into Eq. (17), the first-order recursive equation for solving the displacement response by the effect of structural random factors can be obtained:

Zeroth-order:

25 $$\user2{M\ddot{\overline{\chi }}}\left( t \right) + \user2{\overline{C}\dot{\overline{\chi }}}\left( t \right) + \left( {\overline{\user2{K}}_{l} + \overline{\user2{K}}_{n} } \right)\overline{\user2{\chi }}\left( t \right) = \overline{\user2{Q}}\left( t \right)$$

First-order:

26 $$\user2{M\ddot{\overline{\chi }}}_{{\Theta_{i} }} \left( t \right) + \user2{\overline{C}\dot{\overline{\chi }}}_{{\Theta_{i} }} \left( t \right) + \left( {\overline{\user2{K}}_{l} + \overline{\user2{K}}_{n} } \right)\overline{\user2{\chi }}_{{\Theta_{i} }} \left( t \right) = \overline{\user2{Q}}_{{{1}\Theta_{i} }} \left( {\overline{\user2{\chi }},t} \right){, (}i = 1, \ldots ,q)$$

where

$$\overline{\user2{Q}}_{{{1}\Theta_{i} }} \left( {\overline{\user2{\chi }},t} \right) = \overline{\user2{Q}}_{{\Theta_{i} }} \left( t \right) - \left[ {\begin{array}{*{20}c} {\overline{\user2{C}}_{{\Theta_{i} }} \dot{\overline{\chi }}\left( t \right) + } \\ {\left( {\left( {\overline{\user2{K}}_{l} } \right)_{{\Theta_{i} }} + \left( {\overline{\user2{K}}_{n} } \right)_{{\Theta_{i} }} } \right)\overline{\chi }\left( t \right)} \\ \end{array} } \right]{, }\left( {i = 1, \ldots ,q} \right)$$

The expression of the displacement’s mathematical expectation and the displacement covariance can be described as

27 $$\begin{array}{*{20}c} {E\left[ {\varvec{\chi}} \right] = \int_{ - \infty }^{\infty } {{\varvec{\chi}}\left( {{\varvec{\varTheta}},t} \right)} f\left( {\varvec{\chi}} \right)d{\varvec{\varTheta}}} \\ {Cov\left( {\chi_{i} ,\chi_{j} } \right) = \int_{ - \infty }^{\infty } {\left( {\chi_{i} - \overline{\chi }_{i} } \right)} \left( {\chi_{j} - \overline{\chi }_{j} } \right)f\left( {\varvec{\chi}} \right)d{\varvec{\chi}}} \\ \end{array}$$

The aeroelastic displacement expectation and covariance with first order accuracy can be obtained by substituting Eq. (22) into Eq. (27):

28 $$\begin{array}{*{20}c} {E\left[ {\varvec{\chi}} \right] = \overline{\user2{\chi }}} \\ {Cov\left( {\overline{\chi }_{i} , \, \overline{\chi }_{j} } \right) = \sum\limits_{l = 1}^{q} {\sum\limits_{k = 1}^{q} {\overline{\chi }_{{i\Theta_{k} }} \overline{\chi }_{{j\Theta_{l} }} Cov\left( {\Theta_{k} ,\Theta_{l} } \right)} } } \\ \end{array}$$

From Eqs. (25–28), the expectation and covariance of displacement can be obtained by calculating the differential equation $q + 1$ times. However, they only have first-order accuracy. For the purpose of improving accuracy, Eqs. (22–26) are extended to the second order:

29 $${\varvec{\chi}}\left( {{\varvec{\varTheta}},t} \right) = \overline{\user2{\chi }}\left( t \right) + \sum\limits_{i = 1}^{q} {\overline{\user2{\chi }}_{{\Theta_{i} }} \left( t \right)} \Delta \Theta_{i} + \frac{1}{2}\sum\limits_{i,j = 1}^{q} {\overline{\user2{\chi }}_{{\Theta_{i} \Theta_{j} }} \left( t \right)} \Delta \Theta_{i} \Delta \Theta_{j}$$

30 $$\begin{array}{*{20}c} \begin{gathered} {\varvec{M}}\left( {\user2{\Theta , }t} \right) = \overline{\user2{M}}\left( t \right) + \sum\limits_{i = 1}^{q} {\overline{\user2{M}}_{{\Theta_{i} }} \left( t \right)} \Delta \Theta_{i} + \frac{1}{2}\sum\limits_{i,j = 1}^{q} {\overline{\user2{M}}_{{\Theta_{i} \Theta_{j} }} \left( t \right)} \Delta \Theta_{i} \Delta \Theta_{j} \hfill \\ {\varvec{C}}\left( {\user2{\Theta , }t} \right) = \overline{\user2{C}}\left( t \right) + \sum\limits_{i = 1}^{q} {\overline{\user2{C}}_{{\Theta_{i} }} \left( t \right)} \Delta \Theta_{i} + \frac{1}{2}\sum\limits_{i,j = 1}^{q} {\overline{\user2{C}}_{{\Theta_{i} \Theta_{j} }} \left( t \right)} \Delta \Theta_{i} \Delta \Theta_{j} \hfill \\ \end{gathered} \\ {{\varvec{K}}_{l} \left( {\user2{\Theta , }t} \right) = \overline{\user2{K}}_{l} \left( t \right) + \sum\limits_{i = 1}^{q} {\left( {\overline{\user2{K}}_{l} } \right)_{{\Theta_{i} }} \left( t \right)} \Delta \Theta_{i} + \frac{1}{2}\sum\limits_{i,j = 1}^{q} {\left( {\overline{\user2{K}}_{l} } \right)_{{\Theta_{i} \Theta_{j} }} \left( t \right)} \Delta \Theta_{i} \Delta \Theta_{j} } \\ {{\varvec{K}}_{n} \left( {\user2{\Theta , }t} \right) = \overline{\user2{K}}_{n} \left( t \right) + \sum\limits_{i = 1}^{q} {\left( {\overline{\user2{K}}_{n} } \right)_{{\Theta_{i} }} \left( t \right)} \Delta \Theta_{i} + \frac{1}{2}\sum\limits_{i,j = 1}^{q} {\left( {\overline{\user2{K}}_{n} } \right)_{{\Theta_{i} \Theta_{j} }} \left( t \right)} \Delta \Theta_{i} \Delta \Theta_{j} } \\ {{\varvec{Q}}\left( {{\varvec{\varTheta}},t} \right) = \overline{\user2{Q}}\left( t \right) + \sum\limits_{i = 1}^{q} {\overline{\user2{Q}}_{{\Theta_{i} }} \left( t \right)} \Delta \Theta_{i} + \frac{1}{2}\sum\limits_{i,j = 1}^{q} {\overline{\user2{Q}}_{{\Theta_{i} \Theta_{j} }} \left( t \right)} \Delta \Theta_{i} \Delta \Theta_{j} } \\ \end{array}$$

As seen in Eq. (24), due to the influence of geometric nonlinearity, the first partial derivative of stiffness matrix with respect to random variable has a complex form, as the matter of the fact that second derivative will be more complicated, exact form of the second partial derivative is not given here. Where, $\overline{\user2{\chi }}_{{\Theta_{i} \Theta_{j} }} \left( t \right)$ expressed as the second partial derivative of $\overline{\user2{\chi }}\left( t \right)$ with respect to $\Theta_{i}$ and $\Theta_{j}$ estimated at $\overline{\user2{\Theta }}$. The terms in Eq. (22) have the same meaning. Then, combined with Eq. (17), the second order recursive equation will be obtained:

Zeroth-order:

31 $$\user2{M\ddot{\overline{\chi }}}\left( t \right) + \user2{\overline{C}\dot{\overline{\chi }}}\left( t \right) + \left( {\overline{\user2{K}}_{l} + \overline{\user2{K}}_{n} } \right)\overline{\user2{\chi }}\left( t \right) = \overline{\user2{Q}}\left( t \right)$$

First-order:

32 $$\user2{M\ddot{\overline{\chi }}}_{{\Theta_{i} }} \left( t \right) + \user2{\overline{C}\dot{\overline{\chi }}}_{{\Theta_{i} }} \left( t \right) + \left( {\overline{\user2{K}}_{l} + \overline{\user2{K}}_{n} } \right)\overline{\user2{\chi }}_{{\Theta_{i} }} \left( t \right) = \overline{\user2{Q}}_{{{1}\Theta_{i} }} \left( {\overline{\user2{\chi }},t} \right) \, i = 1, \ldots ,q$$

where

33 $$\overline{\user2{Q}}_{{{1}\Theta_{i} }} \left( {\overline{\user2{\chi }},t} \right) = \overline{\user2{Q}}_{{\Theta_{i} }} \left( t \right) - \left[ {\begin{array}{*{20}c} {\overline{\user2{C}}_{{\Theta_{i} }} \dot{\overline{\chi }}\left( t \right) + } \\ {\left( {\left( {\overline{\user2{K}}_{l} } \right)_{{\Theta_{i} }} + \left( {\overline{\user2{K}}_{n} } \right)_{{\Theta_{i} }} } \right)\overline{\chi }\left( t \right)} \\ \end{array} } \right] \, i = 1, \ldots ,q$$

Second-order:

34 $$\user2{M\ddot{\hat{\chi }}}_{2} \left( t \right) + \user2{\overline{C}\dot{\hat{\chi }}}_{2} \left( t \right) + \left( {\overline{\user2{K}}_{l} + \overline{\user2{K}}_{n} } \right)\hat{\user2{\chi }}_{2} \left( t \right) = {\varvec{Q}}_{2} \left( {\overline{\user2{\chi }}{,}\overline{\user2{\chi }}_{{\Theta_{i} }} ,t} \right) \, i = 1, \ldots ,q$$

where

35 $$\begin{aligned} \user2{Q}_{2} \left( {\user2{\bar{\chi },\bar{\chi }}_{{\Theta _{i} }} ,t} \right) &= \sum\limits_{{i,j = 1}}^{n} {\left\{ {\left[ {\tfrac{1}{2}\user2{\bar{Q}}_{{\Theta _{i} \Theta _{j} }} \left( t \right)} \right]Cov\left( {\Theta _{i} ,\Theta _{j} } \right)} \right\}} \\ & \quad- \sum\limits_{{i,j = 1}}^{n} {\left\{ {\left[ \begin{gathered} \tfrac{1}{2}\user2{\bar{C}}_{{\Theta _{i} \Theta _{j} }} \dot{\bar{\chi }}\left( t \right) + \frac{1}{2}\left( {\left( {\user2{\bar{K}}_{b} } \right)_{{\Theta _{i} \Theta _{j} }} + \left( {\user2{\bar{K}}_{g} } \right)_{{\Theta _{i} \Theta _{j} }} } \right)\bar{\chi }\left( t \right) \hfill \\ + \user2{\bar{C}}_{{\Theta _{i} }} \dot{\bar{\chi }}_{{\Theta _{i} }} \left( t \right) + \left( {\left( {\user2{\bar{K}}_{b} } \right)_{{\Theta _{i} }} + \left( {\user2{\bar{K}}_{g} } \right)_{{\Theta _{i} }} } \right)\bar{\chi }_{{\Theta _{i} }} \left( t \right) \hfill \\ \end{gathered} \right]Cov\left( {\Theta _{i} ,\Theta _{j} } \right)} \right\}} \\ \user2{\hat{\chi }}_{2} \left( t \right) = & \frac{1}{2}\sum\limits_{{i,j = 1}}^{n} {\bar{\chi }_{{\Theta _{i} \Theta _{j} }} \left( t \right)Cov\left( {\Theta _{i} ,\Theta _{j} } \right)} \\ \end{aligned}$$

After solving $\hat{\user2{\chi }}_{2} \left( t \right)$ by Eqs. (31–35), the displacement expectation with second order accuracy could be indicated as:

36 $$E\left[ {\varvec{\chi}} \right] = \overline{\user2{\chi }} + \frac{1}{2}\sum\limits_{i = 1}^{n} {\overline{\user2{\chi }}_{{\Theta_{i} \Theta_{j} }} Var\left( {\Theta_{i} } \right)}$$

To check the effectiveness of the results, in the following section, we introduce the classical Monte Carlo method for comparison. Furthermore, the influence of random factors on aeroelasticity is analyzed.

## Numerical simulation analysis

In order to confirm the effectiveness of the theory derived in the study above, and to analyze the effect of the random factors on aeroelasticity, numerical simulations are carried out in this part. In Sect. 5.1, method compare results with available literatures are given. As MCS method is the most common method for a probabilistic analysis^36^, scholars often use MCS to check the validity of SFEM^37, 38^. In Sect. 5.2, We take MCS method to check the statistical characteristics of the displacement. In Sect. 5.3, the displacement response is studied on the basis of the correlation characteristics.

### Verification of the model

In order to verify the fidelity of the model in this paper, the methods of literatures^5, 39, 40^ are used for comparing. The simulation data^5^ are shown in Table 1.

**Table 1:** Simulation parameters.

Semi-span	16 m
Chord	1 m
Angle of attack	2 deg
Elastic axis	0.5 c
Center of gravity	0.5 c
Mass/span	0.75 kg/m
bending stiffness	2.0e4 N.m^2^
Torsional stiffness	1.0 e4 N.m^2^

**Figure 4:**
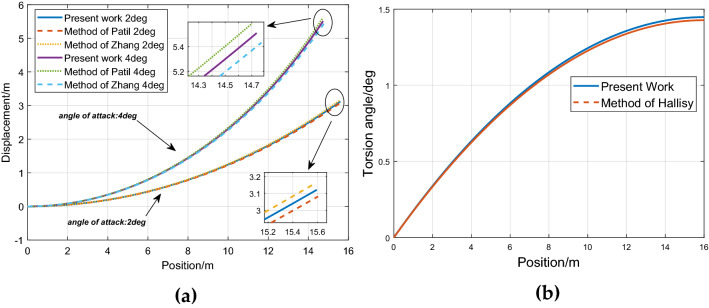
Model validation comparing with the literatures: (**a**) Bending displacement compare results; (**b**) Torsional displacement compare results.

Based on these simulation data, Static deflections of the clamped cantilevered wing is simulated and compared with references. Figure 4a shows the curves under different angles of attack of bending displacement compared with references^5, 39^, Fig. 4b shows the torsional displacement compared with the literature^40^.

Simulation results show that the model presented in this paper can describe the dynamic behavior of the wing well in a nonlinear large deformation case, and the model shows good agreement with literature results.

### Simulation result verification by MCS

Simulation data is shown in Table 2. Geometric properties, structural properties and flight conditions data is provided. In these simulations, the number of elements is 1000, time step is 0.01 s, and Newmark-Beta algorithm is adopted to study the dynamic response of HARW under the action of aerodynamic force and aerodynamic torque caused by initial root angle of attack 2 deg. The aerodynamics is modeled in chapter 4.

**Table 2:** Simulation data.

Semi-span	10 (m)
Chord	1 (m)
Angle of attack	2 (deg)
Elastic axis	0.3 c
Center of gravity	0.5 c
Airfoil data	Flat plate
Mass/Span	0.75 (${\text{kg}}/{\text{m}}$)
bending stiffness	$1.2 \times 10^{7}$(${\text{N}} \cdot {\text{m}}^{{2}}$)
Torsional stiffness	$2.2 \times 10^{7}$(${\text{N}} \cdot {\text{m}}^{{2}}$)
Coefficient of variation of elastic modulus and torsional stiffness	0.03
Flight altitude	20 (km)
Flow speed	25 (m/s)
Atmospheric density	0.0889 (${\text{kg/m}}^{3}$)

Considering $\tilde{E}\left( x \right)$ and $\widetilde{GJ}\left( x \right)$ as random fields, the simulation comparison results of MCS method and SFEM method are shown in Fig. 4. In this section, the random factors are considered to be independent of each other.

**Figure 5:**
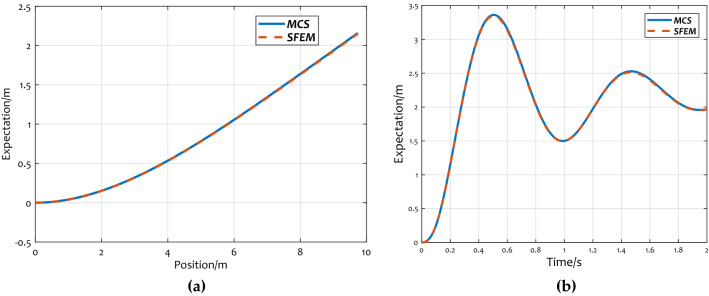
Comparison of displacement expectations: (**a**) displacement at different locations; (**b**) displacement expectations at different time.

In Fig. 5, the solid curves represent the displacement expectations solved by MCS, lines of dashes represent the expected values of displacement calculated by the SFEM proposed in this paper. Figure 5a shows the comparison of expected values of displacement at different locations of the wing when the time is 1 s. Figure 5b displays the comparisons of the vertex displacement expectations at different time points. As seen in the figures, the two curves overlap well, which indicates that the aeroelastic model deduced by SFEM in this study has high reliability.

**Figure 6:**
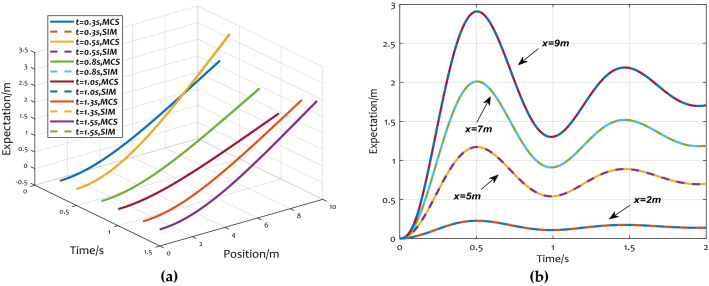
Displacement expectations between MCS and SFEM: (**a**) displacement expectations at different time; (**b**) node displacements at different positions.

To further check the validity of the SEFM aeroelastic model, Fig. 6a shows the comparison of displacement expectations of each position at the simulation time of 0.5, 0.8, 1.0 s, 1.3 s, 1.5 s, and Fig. 6b shows the comparison of node displacements at positions of 2, 5, 7, and 9 m at different times. In Fig. 6b, the solid curves are obtained by MCS, and the dashed curves are calculated by SFEM, respectively.

**Figure 7:**
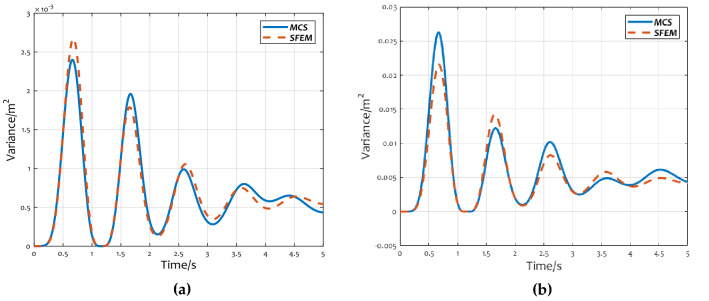
Comparison of displacement variance: (**a**) variance of displacement at 5 m; (**b**) variance of displacement at 10 m.

Figure 7a,b show the variance curves of displacement at 5 m and 10 m with time, respectively. The solid and dashed lines represent the results of MCS and SFEM respectively. As can be seen from Fig. 7, the displacement variance of SFEM has certain discrepancies with MCS method, which is because the variance derived in this paper has only first-order accuracy. However, the comparison results imply that model presented in this paper could well reflect the displacement variance.

After further comparing the calculation results of displacement expectation and variance, it is found that the calculation error of variance is greater than expectation. The reason for this is that the variance results derived in this paper have only first order accuracy.

**Figure 8:**
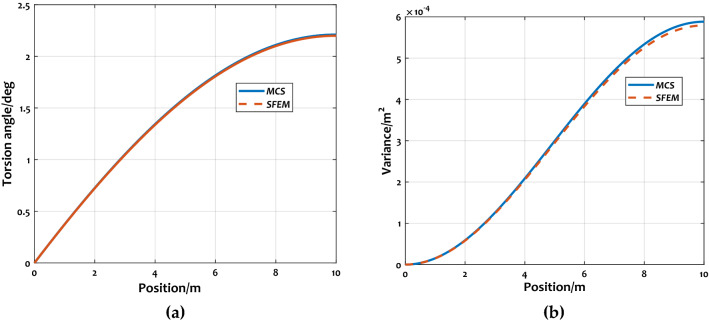
Comparison of statistical characteristics of wing torsion angle: (**a**) torsion angle expectations; (**b**) torsion angle variances.

The curves of expectations and variances of wing torsion angle can be seen in Fig. 8a,b. From the simulation results, it can be seen that the method in this study could well predict the statistical characteristics of torsion angle. Similar to the bending displacement, the accuracy of displacement expectation is better than the variance.

The above simulation is used to compare the displacement expectations with second-order accuracy. However, from the expression of the second-order recursive equation and the simulation calculation, it can be concluded that solving the displacement expectation with higher accuracy needs a large amount of calculation. In particular, it is necessary to calculate the second-order partial derivatives of damping matrix and stiffness matrix for random variables. In contrast, the first order recursive equation has a relatively small amount of calculation. In order to explore the accuracy of the calculation results of the first-order recursive equation, Table 3 shows the comparisons of the first-order and second-order recursive equations.

**Table 3:** Comparison of first-order and second-order accuracy displacement expectation.

Position ($m$)	MCS	Second-order accuracy ($m$)	relative error	First-order accuracy ($m$)	relative error
2.0	0.15009528	0.15064420	0.0037	0.15159623	0.0100
4.0	0.52280123	0.52471065	0.0037	0.52855204	0.0110
6.0	1.02166840	1.02539581	0.0036	1.03188508	0.0100
8.0	1.59210307	1.58363042	0.0053	1.57299783	0.0120
10.0	2.15014257	2.15797714	0.0036	2.17164399	0.0100

It can be seen from Table 3, that when the coefficient of variation of random variables is 0.03, the accuracy of the results obtained by the second-order recursive equation is better than that of the first-order recursive equation. However, the first-order recursive equation can also predict the displacement statistical characteristics well when the coefficient of variation is small. Moreover, with relatively small amount of computation, the first order recursive equation is still competitive.

### Characteristic analysis of displacement response with random field correlation

This section studies the impacts of random variables with diverse correlations on aeroelastic response based on the parameters of Table 2. Firstly, the correlation between random field and discrete random variables is studied. However, in this section, it is necessary to note that it is assumed that the elastic modulus random field and the torsional stiffness random field have the same correlation.

**Figure 9:**
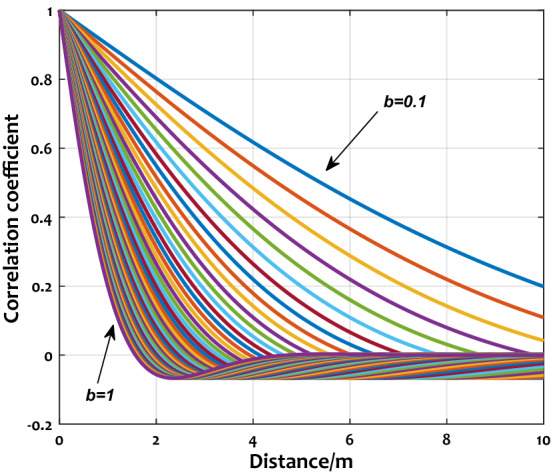
Relationships between $b$, $\zeta$, and $\tau$.

In Fig. 9, the relationship between parameters $b$, $\zeta$, and the correlation of random fields are shown. When $b$ is a certain value, $\tau \left( \zeta \right)$ decreases with the increment of the distance between two variables. On the other hand, the increase of $b$ will cause the decrease of the correlation. Moreover, when $b$ increases to a certain value, the correlation of random fields will decrease sharply with the increase of distance. The variance characteristics of random variables obtained will be affected by the variation of correlation characteristics of random fields. Thus the aeroelastic effect of the wing will be further influenced.

**Figure 10:**
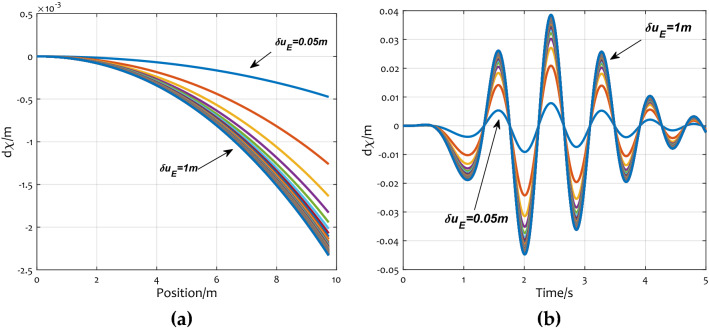
The impact of random field correlation: (**a**) the change of $d{\varvec{\chi}}$ at 1.3 s by different $\delta u$; (**b**) $d{\varvec{\chi}}$ response at different time by different $\delta u$.

Let $d{\varvec{\chi}} = \sum\limits_{i = 1}^{n} {\overline{\user2{\chi }}_{{\Theta_{i} \Theta_{j} }} Var\left( {\Theta_{i} } \right)}$, $d{\varvec{\chi}}$ denotes that the calculated results of the second-order recursive equations compensate the mean displacement obtained by the first-order recursive equations. Increased accuracy brought by its existence. When taking different correlation distances, the influence on $d{\varvec{\chi}}$ is shown in Fig. 10.

When the correlation distance $\delta u$ changes from 0.05 m to 1 m, the curves in Fig. 10a show the changes of $d{\varvec{\chi}}$ at the 1.3 s. The variation of the displacement with time is shown in Fig. 10b. As shown in the figure, the curves are the $d{\varvec{\chi}}$ response trends of $\delta u = 0.05,0.06,0.07,0.08, \ldots ,1 \, m$. $d{\varvec{\chi}}$ is positively correlated with the changing trend of *δu*. Since $\overline{\user2{\chi }}$ has nothing to do with random variables, as the increases of $\delta u$, so does the expected displacement $E\left[ {\varvec{\chi}} \right]$. The simulations above show that the larger the $\delta u$, the stronger the correlation. That is, the greater the correlation, the larger the value of $d{\varvec{\chi}}$.

From Fig. 10a, As $\delta u$ gets bigger, the rate of change of $d{\varvec{\chi}}$ gets smaller. That is to say, when $\delta u$ increased to be large enough, $d{\varvec{\chi}}$ converges to a definite value.

**Figure 11:**
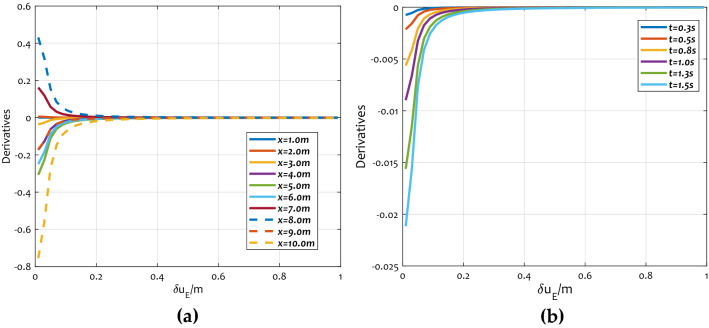
Correlation analysis between $d{\varvec{\chi}}$ and $\delta u$: (**a**) $d{\varvec{\chi}}$ to $\delta u$ curves of different position; (**b**) $d{\varvec{\chi}}$ to $\delta u$ curves of different time.

Figure 11 shows the derivative trend curves $d{\varvec{\chi}}$ to $\delta u$, which can explain the conclusion in Fig. 11 more intuitively. With the increase of $\delta u$, the derivative curves of $d{\varvec{\chi}}$ to $\delta u$ trend to 0.

The reason for this phenomenon is that the random field correlation and variance reduction function will not change all the time. As $\delta u$ goes up, the random field correlation, as well as the variance reduction function gradually be stabilized. In addition, according to the analysis above, the larger the $\delta u$ is, the more the displacement compensation is. That is to say, the displacement deviation of the first order recursive equation increases as the correlation increases. Since $\overline{\user2{\chi }}$ is independent of the correlation, the expectation of displacement goes up as the correlation distance increasing. This means that the weaker the correlation, the higher the accuracy of the first order recursive equation.

## Conclusions

In this paper, the aeroelasticity of the flexible High-Aspect-Ratio wing is studied by considering elastic modulus and torsional stiffness as random parameters. The wing is simplified as a cantilevered Eula-Bernoulli beam, and an aeroelastic model is established. Then, the first order and second order recursive stochastic nonlinear finite element equations of wing considering the influence of structural random parameters are derived by using local averaging and perturbation methods. The expression of the numerical characteristics of the aeroelastic response of the wing is obtained. After that, Monte Carlo method is adopted to verify the effectiveness of the method. Conclusions of this paper mainly include:

- Simulations indicate that the proposed method could estimate the statistical characteristics of the wing’s aeroelastic effects accurately.
- Compared with the second order recursive equation, when the coefficient of variation is small, the first order recursive equation not only ensures good accuracy, but also has better computational efficiency.
- The correlation of random structural parameters will affect the aeroelastic displacement response of wing. The stronger the correlation, the worse the accuracy of the first order recursive equation.

The elastic modulus and torsional stiffness of the wing are chosen as the random factors in this study, but there are many random factors in actual structures. In the future work, other random factors (such as material density, structure nonlinear factors, structural load, etc.) will also be considered. On the other hand, how to select the first or second order recursive equation according to the coefficient of variation and the correlation of random structural parameters is worth further study.
